# Allergic Bronchopulmonary Aspergillosis in Asthma and Cystic Fibrosis

**DOI:** 10.1155/2011/843763

**Published:** 2011-04-05

**Authors:** Alan P. Knutsen, Raymond G. Slavin

**Affiliations:** ^1^Department of Pediatrics, Saint Louis University, 1465 South Grand Boulevard, St. Louis, MO 63104, USA; ^2^Division of Infection Diseases, Allergy and Immunology, Saint Louis University, 1465 South Grand Boulevard, St. Louis, MO 63104, USA; ^3^Department of Internal Medicine, Saint Louis University, 1465 South Grand Boulevard, St. Louis, MO 63104, USA

## Abstract

Allergic bronchopulmonary aspergillosis (ABPA) is a Th2 hypersensitivity lung disease in response to *Aspergillus fumigatus* that affects asthmatic and cystic fibrosis (CF) patients. Sensitization to *A. fumigatus* is common in both atopic asthmatic and CF patients, yet only 1-2% of asthmatic and 7–9% of CF patients develop ABPA. ABPA is characterized by wheezing and pulmonary infiltrates which may lead to pulmonary fibrosis and/or bronchiectasis. The inflammatory response is characterized by Th2 responses to *Aspergillus* allergens, increased serum IgE and eosinophilia. A number of genetic risks have recently been identified in the development of ABPA. These include HLA-DR and HLA-DQ, IL-4 receptor alpha chain (*IL-4RA*) polymorphisms, IL-10-1082GA promoter polymorphisms, surfactant protein A2 (SP-A2) polymorphisms, and cystic fibrosis transmembrane conductance regulator gene (*CFTR*) mutations. The studies indicate that ABPA patients are genetically at risk to develop skewed and heightened Th2 responses to *A. fumigatus* antigens. These genetic risk studies and their consequences of elevated biologic markers may aid in identifying asthmatic and CF patients who are at risk to the development of ABPA. Furthermore, these studies suggest that immune modulation with medications such as anti-IgE, anti-IL-4 and/or IL-13 monoclonal antibodies may be helpful in the treatment of ABPA.

## 1. Introduction

Allergic bronchopulmonary aspergillosis (ABPA) is a hypersensitivity lung disease due to bronchial colonization by *Aspergillus fumigatus* that occurs in susceptible patients with asthma and cystic fibrosis (CF). The first published description of ABPA as an entity came from the United Kingdom in 1952 [1], while the first cases in the United States were reported a decade later [2, 3]. ABPA affects approximately 1-2% of asthmatic patients and 7–9% of CF patients [4–6]. If unrecognized or poorly treated, ABPA leads to airway destruction, bronchiectasis, and/or pulmonary fibrosis, resulting in significant morbidity and mortality.

## 2. Biology of *Aspergillus fumigatus*

A number of fungi may lead to allergic bronchopulmonary mycoses (ABPM), but the genus *Aspergillus* contains the predominant organisms causing these pulmonary disorders; *Aspergillus fumigatus* is the species most commonly associated with ABPM. It is a ubiquitous, saprophytic mold found in both outdoor and indoor air, in potting soil, crawl spaces, compost piles, mulches, freshly cut grass, decaying vegetation, and sewage treatment facilities [7, 8]. *A. fumigatus* is found worldwide including the United States, where it is especially prevalent in the Midwest and East coast. *Aspergillus* spores are common indoors and outdoors. *Aspergillus* species are thermotolerant growing at 15° to 53°C temperatures and particularly grows well at 37° to 40°C which allows for sporulation in human bronchi. Septated hyphae of *A. fumigatus* are 7 to 10 *μ*m in diameter with branching at 45° angles. Hyphae may be demonstrated in sputum, mucus plugs or sinus debris with Gomori methenamine-silver or periodic acid-Schiff stains.

## 3. *Aspergillus*-Associated Pulmonary Diseases

*A. fumigatus* is responsible for a variety of pulmonary diseases depending on the individual's genetic status and immunologic responses to *Aspergillus* antigens. These include:

1. atopic asthma,
2. invasive aspergillosis,
3. aspergilloma (mycetoma),
4. hypersensitivity pneumonitis,
5. asthma,
6. allergic bronchopulmonary aspergillosis
  - in asthma and
  - in cystic fibrosis.

### 3.1. Atopic Asthma

Asthmatic patients may develop IgE sensitivity to molds including *A*. *fumigatus*. A number of investigators have reported that onset, persistence, and severity of asthma are associated with sensitivity to fungus, especially *Cladosporium* and *Alternaria* [9–15]. Recently, David Denning's group coined the term severe asthma associated with fungal sensitivity (SAFS) [16]. In their studies, sensitivity to *A. fumigatus *was the most common fungus causing sensitization. Furthermore, treatment with the antifungal agent itraconazole improved their asthma. Another series of studies by Andrew Wardlaw's group also demonstrated that sensitivity to *A. fumigatus* was associated with severe asthma [17]. In those studies, *A. fumigatus* could be cultured from sputum specimens. FEV-1 was decreased and total IgE levels were elevated. The asthmatic patients in both Denning's and Wardlaw's studies did not fulfill criteria for ABPA. Colonization with *A. fumigatus* and development of ABPA also occurs in cystic fibrosis patients [18]. A number of investigators have reported that colonization with *A. fumigatus* in CF is associated with decreased FEV-1 and more rapid pulmonary function decline even when not diagnosed as ABPA. These studies suggest that there is a spectrum of pulmonary disease states due to *A. fumigatus *sensitization in asthmatic and CF patients from severe asthma to ABPA.

### 3.2. Invasive Aspergillosis

Invasive aspergillosis occurs in individuals who have an iatrogenic or congenital immunodeficiency. The congenital immunodeficiencies are neutrophil defects, such as chronic granulomatous disease (CGD) and leukocyte adhesion defects (LAD). The iatrogenic defects are secondary to chemotherapy that results in neutropenia. In invasive aspergillosis, *A. fumigatus* invades the bronchial epithelia resulting in pneumonia, tracheobronchitis, pulmonary diseases, and pleural effusions. *A. fumigatus* may result in osteomyelitis and central nervous system infections. Treatment is with antifungal medications, such as itraconazole, voriconazole, or posaconazole.

### 3.3. Aspergilloma

Aspergilloma or mycetoma occurs in individuals with pre-existing pulmonary diseases, such as bronchocentric carcinoma, cavitary tuberculosis, pulmonary histoplasmosis, cystic fibrosis, sarcoidosis, and bronchiectasis. *Aspergillus* spores germinate in these structures forming a tangled mass of hyphae, termed a fungus ball. Hemoptysis is a common symptom. IgG anti-*Aspergillus* antibodies are usually extremely elevated, but specific IgE anti-*Aspergillus* antibodies are usually not detectable. Treatment consists of antifungal medication and surgical excision of the cavity.

### 3.4. Hypersensitivity Pneumonitis

Hypersensitivity pneumonitis (HP) or extrinsic allergic alveolitis is caused by a variety of antigens, including bird droppings (Pigeon Breeder's disease), murine urine proteins (Laboratory Worker's lung), thermophilic moldy hay (Farmer's lung), *Aspergillus clavatus* moldy brewery barley (Malt Worker's disease), and others. The symptoms in the acute stage include fever, nonproductive cough, and dyspnea. In the subacute stage, the symptoms are malaise, low-grade fevers, nonproductive cough, and dyspnea. In the chronic stage, the symptoms are chronic cough, dyspnea, fatigue, anorexia, and weight loss. Chest radiograph displays micronodular pulmonary infiltrates, ground-glass opacities, or diffuse infiltrates. The immunologic response to the antigen is a Th1 cell-mediated reaction. IgG-specific antibodies are typically extremely elevated. Treatment is removal of the antigen source and corticosteroids.

## 4. Diagnosis and Staging of ABPA

The diagnosis of ABPA is based on clinical and immunologic reactivity to *A. fumigatus*. The minimal criteria required for the diagnosis of ABPA are (1) asthma or cystic fibrosis with deterioration of lung function, for example, wheezing, (2) immediate *Aspergillus* skin test reactivity, (3) total serum IgE ≥1000 IU/mL, (4) elevated *Aspergillus* specific IgE and IgG antibodies, and (5) chest radiographic infiltrates (Table 1). Additional criteria may include peripheral blood eosinophilia, *Aspergillus* serum precipitating antibodies, central bronchiectasis, and *Aspergillus* containing mucus plug production [19–23]. The designation of ABPA-seropositive (ABPA-S) may be used to classify asthmatic patients who meet the required criteria but lack the proximal or central bronchiectasis (ABPA-CB). The clinician should note that the development of ABPA is not dependent on asthma severity. The diagnosis of ABPA in CF is more complicated and disagreement exists in the literature regarding the diagnostic criteria. The difficulty lies in the fact that the usual criteria for ABPA and the common signs and symptoms of CF overlap. The most recent Cystic Fibrosis Foundation Consensus Conference proposed the following diagnostic criteria: (1) acute or subacute pulmonary deterioration not attributable to another etiology, (2) total serum IgE > 1000 IU/mL, (3) immediate cutaneous reactivity to *Aspergillus* or *in vitro* specific IgE antibodies to *Aspergillus*, and (4) one of the following: *Aspergillus* serum precipitins, elevated specific IgG anti-*Aspergillus* antibodies, or new or recent chest radiographic or chest CT abnormalities that have not cleared with antibiotics and chest physiotherapy (Table 2) [23].

### 4.1. Clinical Staging

The spectrum of ABPA varies widely, from individuals with mild asthma and occasional episodes of pulmonary eosinophilia with no long-term sequelae, to patients with fibrosis, honey-comb lung, and respiratory failure. Patterson and colleagues [22, 23] have suggested a clinical classification with five clinical stages of ABPA in asthmatics (Table 3). Stage I is the initial acute stage of ABPA with many of the typical features of the disease. In stage II, the disease goes into remission; the infiltrates clear, symptoms are reduced, and the serum IgE value will decline by up to 35% within 6 weeks. Stage III is an exacerbation associated with the recurrence of the initial symptoms and a twofold increase in serum IgE levels. Stage IV is reached when patients need continuous corticosteroids either to control their asthma or to prevent a recurrence of ABPA. Stage V is the fibrotic stage, which is present when there is severe upper lobe fibrosis present on the chest radiograph, and it may be associated with honeycombing. The stage V lesions may not respond to corticosteroids, although steroids are often necessary to maintain a bronchodilator response, and severe wheezing may develop if steroids are discontinued. Pulmonary fibrosis is an advanced complication that can lead to pulmonary hypertension and cor pulmonale.

### 4.2. Radiographic Findings

There are several characteristic radiographic abnormalities associated with ABPA [19–23]. The most common lesion is a large, homogeneous shadow in one of the upper lobes with no change in volume. The shadow may be triangular, lobar, or patchy, and it frequently moves to another site. “Tram-line” shadows are fine parallel lines radiating from the hila that represent inflammation of airway walls. Mucoid impaction causes toothpaste shadows or gloved-finger shadows. Adult patients have been reported with normal chest radiographs so radiographic abnormalities are not invariably present. In these individuals, cylindrical bronchiectasis was demonstrated by tomography or CT scan. However, central bronchiectasis is a common complication and finding in all CF patients.

### 4.3. Laboratory Investigations

Laboratory tests that support the diagnosis of ABPA are those that demonstrate allergy to the *A. fumigatus*, such as elevated specific IgE anti-*Aspergillus* antibodies and positive *Aspergillus* precipitins [19–23]. The precipitins are only weakly positive compared with the strong reactions seen in patients with mycetomas. Culture of *A. fumigatus* from the sputum is only a secondary criterion for the diagnosis of ABPA, because a large proportion of individuals with CF without ABPA have *Aspergillus* on sputum cultures. Some normal individuals and many individuals with lung diseases have small numbers of spores in their sputum; these are probably present because of passive inhalation. The presence of hyphae is more specific, and the presence of eosinophils in association with hyphal elements is suggestive of the diagnosis. The presence of eosinophilia in sputum or blood is suggestive of ABPA in asthmatics and is a primary diagnostic criterion. The peripheral blood eosinophil count is usually greater than 1000/mm^3^, and values greater than 3000/mm^3^ are common. As denoted in Table 2, eosinophilia is not a diagnostic criteria of ABPA in CF patients. In the authors' experience, eosinophilia is an uncommon finding in CF ABPA patients.

An increased total serum IgE level is very characteristic of ABPA, and values may reach as high as 30,000 IU/mL. Usually, the level is greater than 1000 IU/mL. Much of the IgE is not specific to *Aspergillus* but is the result of polyclonal B-cell activation. The IgE level is a very useful marker of disease activity, and it can be used to follow outpatients for “flares”. The simple skin-prick test is a useful screening test, as ABPA is very unlikely in patients with a negative reaction. A dual-reaction skin test with an immediate (10–15 minutes) and a late (4–8 hours) reaction occurs in one third of patients with ABPA. Alternatively, serum may be measured for the presence of specific IgE and IgG antibodies. Patients with *Aspergillus*-sensitive asthma will generally have elevated *Aspergillus*-specific IgE antibodies, but patients with ABPA will have much higher *Aspergillus*-specific IgE levels. Hemmann et al. [24] reported that ABPA and *Aspergillus*-sensitive patients have elevated IgE antibodies to recombinant *Aspergillus* Asp f1, Asp f3, Asp f4, and Asp f6 allergens, and that IgE levels to Asp f4 and Asp f6 is highly specific for ABPA in CF patients. 

Differentiating between a bacterial flare versus an ABPA flare in CF patients may be difficult. A useful serum biologic marker may be thymus and activation-regulated chemokine (TARC) or CCL17. Latzin et al. [25] and Hartl et al. [26] reported that TARC was elevated in CF patients with ABPA and was further elevated during acute flares of ABPA. TARC is a chemokine whose ligand is CCR4 receptor on CD4^+^ Th2 cells. 

In ABPA immunoelectrophoresis generally shows one to three precipitin lines, often to only one extract [19–23]. Patients with aspergilloma will have multiple precipitin lines to all antigen extracts. Extracts of *A. fumigatus* contain a complex mixture of proteins that are mainly derived from the hyphae. Antigenic composition varies between batches according to the culture conditions, even within the same laboratory. There is, therefore, a lack of standardization that makes it difficult to compare results between laboratories. However, there has been some success with purification of the major antigenic components that may lead eventually to improved diagnosis.

## 5. Pulmonary Pathology of ABPA

The gross pathology of ABPA demonstrates cylindrical bronchiectasis of the central airways, particularly those to the upper lobes [19–23]. These airways may be occluded by “mucoid impaction,” a condition in which large airways are occluded by impacted mucus and hyphae. Airway occlusion may lead to atelectasis of a segment or lobe and, if the atelectasis is long-standing, saccular bronchiectasis may result. Typically, ABPA is worse in the upper lobes than in the lower lobes. Microscopic examination of the airways shows infiltration of the airway wall with eosinophils, lymphocytes, and plasma cells. The airway lumen may be occluded by mucus containing hyphal elements and inflammatory cells, especially eosinophils. Squamous metaplasia of the bronchial mucosa commonly develops, and granulomas may form. Rarely, bronchiolitis obliterans or bronchocentric granulomatosis develops.

## 6. Immunopathogenesis of ABPA

**Figure 1:**
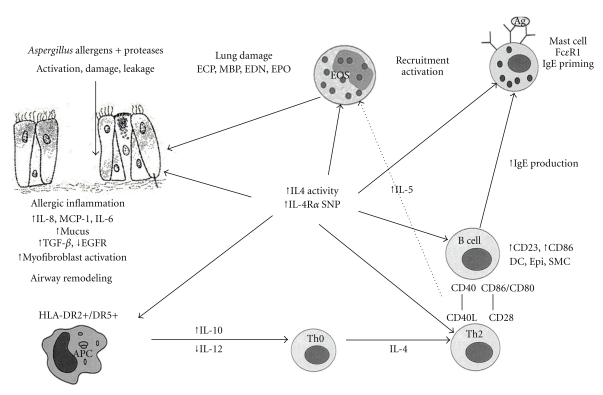
Proposed immunopathogenesis of ABPA. In the pathogenesis of ABPA, *A. fumigatus* proteases have a direct effect on bronchial epithelia causing epithelial cell damage with subsequent stimulation of cytokines and chemokines. *Aspergillus* proteins are processed via HLA-DR2/DR5 bearing dendritic cells that skew the Th0 response to a Th2 response. Th2 cytokines stimulate IgE synthesis and eosinophil activation. This leads to an eosinophilic inflammatory in the bronchial airways. Abbreviations: Af: Aspergillus fumigates, Asp fx: *Aspergillus fumigatus* proteins, APC: antigen presenting cell, MBP: major basic protein, ECP: eosinophil cationic protein, EDN: eosinophil derived neurotoxin, VLA: very late activation antigen, VCAM: vascular cell adhesion molecule, CxCR and CCR: chemokines receptors, MCO: monocyte chemotactic protein, sCD23: soluble CD23, cyst-LT: cysteinyl leukotriene.

As seen in Figure 1, the pathogenesis of ABPA in susceptible persons begins with the inhalation of *A. fumigatus* spores that germinate into hyphae deep within the bronchi. Fragments of hyphae have also been found within the lung parenchyma, potentially resulting in high concentrations of *Aspergillus* allergens exposed to the respiratory epithelium and immune system [7, 27–29]. These allergens are processed by HLA-DR2 or HLA-DR5 bearing antigen presenting cells (APC) and presented to T cells within bronchoalveolar lymphoid tissue (BALT). The resulting CD4^+^ T cell responses to *Aspergillus* are skewed toward Th2 response with the production of IL-4, IL-5, and IL-13 cytokines. 

### 6.1. Effect of *Aspergillus* on Bronchial Epithelium

*A. fumigatus* spores 3 to 5 *μ*m in size are inhaled and germinate deep within the bronchi into hyphae [27]. In addition, fragments of the hyphae can be identified within the interstitial of the pulmonary parenchyma. The implication of this is that there is the potential for high concentrations of *A. fumigatus* allergens exposed to the respiratory epithelium and immune system. *A. fumigatus* releases a variety of proteins, including superoxide dismutases, catalases, proteases, ribotoxin, phospholipases, hemolysin, gliotoxin, phthioic acid, and other toxins. The first line of defense against *Aspergillus* colonization in the lungs is macrophage and neutrophil killing of the conidia and the hyphae. In the development of ABPA, Kauffman's group proposed that *Aspergillus *proteins have a direct effect on the pulmonary epithelia and macrophage inflammation [30, 31]. They demonstrated that *Aspergillus* proteases induce epithelial cell detachment. In addition, protease-containing culture filtrates of *Aspergillus* induce human bronchial cell lines to produce proinflammatory chemokines and cytokines, such as IL-8, IL-6, and MCP-1. Thus, various *Aspergillus* proteins have significant biologic activity that disrupts the epithelial integrity and induces a monokine inflammatory response. This protease activity allows for enhanced allergen exposure to the bronchoalveolar lymphoid tissue immune system. This is evident by the bronchoalveolar lymphoid tissue synthesis of *Aspergillus*-specific IgE and IgA antibodies.

An important pathogenic feature of *Aspergillus* and other microbes is their ability to interact with epithelial cells on the mucosal surface. Macrophage and neutrophil killing of the conidia and hyphae is the first line of defense against colonization in the lungs [32–35]. This is evidenced by an increased susceptibility to invasive pulmonary aspergillosis in patients with chronic granulomatous disease, a disorder of phagocyte killing. *A. fumigatus* has several virulence factors, including proteolytic enzymes that can interfere with humoral and cellular defense in the airways [36, 37]. Proteases from *Aspergillus* and other fungi, including *Alternaria* and *Cladosporium*, have been shown to cause epithelial cell detachment, though *Aspergillus* proteases demonstrated more activity at lower concentrations [36–39]. 

In addition to damaging the integrity of the epithelial cell layer, Kauffman's group demonstrated that protease containing culture infiltrates of *A. fumigatus* induced human bronchial cell lines to produce proinflammatory chemokines and cytokines, such as monocyte chemoattractant protein (MCP)-1, IL-8, and IL-6 [30]. MCP-1 has been implicated in directly stimulating the development of Th2 cells [40]. The cytokine-release activity could be ascribed to the proteolytic activities of these extracts [30, 38]. These observations suggested that proteolytic enzymes released by *Aspergillus*, growing on and between epithelial cells, were responsible for the induction of chemoattractive cytokines by epithelial cells and the corresponding inflammation. It was proposed that induction of the severe inflammatory responses by the direct activation of epithelial cells may cause additional harm to the epithelial cell layer [36]. Destruction of the epithelial cell barrier either by fungal proteases or eosinophilic and neutrophilic inflammation was followed by repair mechanisms, resulting in the influx of serum proteins and extracellular matrix proteins to the luminal side of the epithelium [41]. Because spores and mycelium of *A. fumigatus* have surface structures that are able to interact with extracellular matrix molecules, damage and repair mechanisms of the airway mucosa may facilitate the binding of *Aspergillus* to the damaged sites of the airways. The enhanced release of proteolytic enzymes and allergens on the epithelial surface would induce a continuous inflammatory response and mast cell degranulation, resulting in severe and long-lasting periods of exacerbations of ABPA.

### 6.2. *Aspergillus*-Specific Th2 Cells

The immune response to *Aspergillus* antigens in ABPA patients, as well as allergic asthmatic and CF patients, is characterized by a Th2 CD4^+^ T lymphocyte response [27, 42–46]. Skin test reactivity to *Aspergillus* is found in 20%–25% of asthmatic patients [5, 47, 48] and 31%–59% of CF patients [24, 49]. Although sensitization is common in these populations, only a small percentage of patients develop ABPA.

Several groups have observed T cell lymphoproliferative responses to crude *Aspergillus* extracts [42, 50–52]. Subsequently, *Aspergillus*-specific T cell responses were examined and shown to enhance B cell IgE synthesis [52]. In addition, Asp f1 T cell lines were generated, and the phenotypes were found to be CD4^+^ CD25^+^ T cells with the cytokine profile IL-4^+^ and IFN*γ* ^−^, indicating Th2 CD4^+^ T cells [4]. Chauhan et al. [53] subsequently developed T cell clones from asthmatic ABPA patients and demonstrated either Th2 (IL-4^+^, IFN-*γ* ^−^) or Th0 (IL-4^+^, IFN-*γ* ^+^) patterns. We demonstrated that ABPA subjects have increased frequency of IL-4^+^ CD3^+^ T cells from Asp f2/f3/f4 stimulated peripheral blood lymphocytes compared to *Aspergillus* sensitive non-ABPA subjects [4]. IL-4 produced by T lymphocytes binds to the IL-4 receptor (IL-4R) on B cells, and in association with the CD40L/CD40 signals, results in IgE isotype switching and B cell proliferation [54]. IL-4 also increases the expression of CD86, which has been linked to eosinophilic airway inflammation and airway hyperresponsiveness after allergen challenge. A central question then is how ABPA patients differ from *Aspergillus-*sensitive atopic asthmatic and CF patients. We hypothesize that ABPA develops in genetically susceptible individuals with asthma and CF because of increased frequency and/or activity of *A. fumigatus*-specific Th2 CD4^+^ cells. We further propose that polymorphisms of the interleukin-4 receptor alpha chain (*IL-4RA*) subunit and HLA-DR2/DR5 are the genetic susceptibility risk factors responsible for the development of ABPA.

### 6.3. HLA-DR and HLA-DQ

HLA-DR restriction has been shown to be a risk factor for the development of ABPA (Table 4). Chauhan et al. [53, 55, 56] observed that asthmatic and CF patients who expressed HLA-DR2 and/or DR5 but lacked HLA-DQ2 were at increased risk for ABPA after exposure to *A. fumigatus*. Within HLA-DR2 and HLA-DR5, there are restricted genotypes. In particular, HLA-DR2 HLA-DRB1*1501 and HLA-DRB1*1503 genotypes were reported to provide high relative risk. On the other hand, 40% to 44% of non-ABPA atopic *Aspergillus*-sensitive individuals have the HLA-DR2 and/or DR5 type. Further studies indicated that the presence of HLA-DQ2, especially DQB1*0201, provided protection from the development of ABPA. Furthermore, Chauhan et al. [53] demonstrated that Asp f l allergen has a low-affinity of binding to HLA-DR. This is consistent with Th2 T cell response previously reported by others in that strong antigen HLA-DR-Ag-TCR affinity binding favored a Th1 cellular response whereas low-affinity binding favored a Th2 humoral response [57–61]. Four major V*β* chains, V*β* 3, 6, 13, and 14, reacted to Asp f1.

### 6.4. IL-4 Responses in ABPA

Human studies and murine models have shown that CD4^+^ Th2 cells and their cytokines are central to the development of ABPA [4, 43–46, 50, 62]. In particular, IL-4 has a key role in the allergic inflammatory response with effects on various cell populations. Its functions include increasing VCAM-1 expression on endothelial cells, which enhances the recruitment of other immune cells, particularly eosinophils, stimulating proliferation of fibroblasts, important in airway remodeling, and increasing Th2 differentiation while decreasing Th1 differentiation and the production of IFN-*γ* [63, 64]. IL-4 also has a myriad of effects on B lymphocytes including the stimulation of growth and activation, increasing HLA-DR class II expression important for antigen presentation, and inducing cell surface expression of CD23 and soluble CD23. This cell surface molecule is the low-affinity IgE receptor (Fc*ε*RII) and an activation marker present on a number of cells including B cells, activated T cells, monocytes, and eosinophils. CD23 plays a role in augmenting B cell IgE synthesis through its interactions with CD21 [65, 66]. Recently, anti-CD23 monoclonal antibody was administered to atopic asthmatic subjects and resulted in decreased serum IgE levels [67]. In addition, IL-4 has a more direct role in IgE isotype switching by B-cells. It should be noted that IL-13 may also stimulate the synthesis of IgE and is the only other cytokine that has this capability [68–70]. Recently, increased sensitivity to *in vitro* IL-4 stimulation as measured by enhanced expression of the low-affinity IgE receptor (CD23) on B cells was observed in ABPA patients [43, 44]. This was associated with single-nucleotide polymorphisms of the IL-4 receptor alpha chain (*IL-4RA*) in 92% of ABPA subjects, principally the IL-4-binding single-nucleotide polymorphism ile75val [29, 45, 46]. This increased sensitivity to IL-4 is demonstrated by increased expression of CD23 and CD86 on B cells of ABPA subjects and increased CD23 expression during flares of ABPA [29]. CD23 is expressed on a variety of cells, including B cells, natural killer cells, subpopulations of T cells, and a subpopulation of dendritic cells. T-cell CD23 and B-cell CD21 form a costimulatory pathway. T-cell CD28 and B-cells CD80 and CD86 costimulatory pathways activate both T and B cells, and CD28 : CD86 is important in IgE synthesis. CD86 is also found on dendritic cells that have the histamine receptor 2, which skews antigen-specific T cells to a Th2 response. We have also observed increased CD86 expression on monocyte-derived dendritic cells of ABPA subjects. Thus, antigen-presenting cells such as monocytes and dendritic cells bearing HLA-DR2 and/or HLA-DR5 and increased sensitivity to IL-4 stimulation probably play a critical role in skewing *A. fumigatus*-specific Th2 responses in ABPA.

### 6.5. IL-4 Alpha Chain Receptor (*IL-4RA*) Polymorphisms

The IL-4 receptor is a type I cytokine receptor and exists as a heterodimer that shares a subunit, IL-4 receptor alpha chain (*IL-4RA*), with the IL-13 receptor alpha (IL-13RA) [71]. There are two types of IL-4 receptors. Type I receptors, found on all lymphohematopoietic cells, are composed of the *IL-4RA* and the common gamma chain (*γ*C), which is also a component of IL-2, IL-7, IL-9, IL-15, and IL-21 cytokine receptors [72]. IL-4 receptor type II, also known as the IL-13 receptor, is formed by the association of *IL-4RA* with the IL-13RA subunits and is located on immune cells, bronchial epithelium, and vascular endothelium. IL-4 stimulates both type I and type II receptors while IL-13 signals through type II receptors.

A potential gain-of-function in the *IL-4RA* subunit may be responsible for B cell hyper-reactivity in ABPA. As a consequence of increased IL-4R activity, proinflammatory cytokines skew T cell responses to a dominant Th2 pattern which ultimately contributes to the pathophysiology and progression of ABPA. There are eight naturally occurring single nucleotide polymorphisms (SNPs) of the *IL4RA* gene: ile75val, glu400ala, cys431arg, ser436leu, ser503pro, gln576arg, ser752ala, and ser786pro reported thus far [73–83]. Chromosome 16, which has been associated with asthma, contains the *IL-4RA* gene [78]. Studies have identified a number of these SNPs to be associated with atopy prevalence and asthma severity. In 1997 Khurana Hershey et al. [74] initially reported on a high prevalence of atopy and a gain-of-function in the *IL-4RA* as measured by increased CD23 expression in patients with gln576arg and a later study found that this allele correlated with asthma severity [80]. Hershey's group found that the presence of two variants, val75 and arg576 together, resulted in elevated IL-4 dependent CD23 expression which was not observed when these SNPs were present alone [83]. In our studies, the presence of the val75 allele, located within the IL-4 binding region, was found in 87.5% of ABPA subjects examined, while the cytoplasmic SNPs were present much less frequently at 27.3% for ala400, 27.3% pro503, and 27.3% arg576, and 9.1% arg431. Although these alleles, particularly val75, appear to be common in the general population, their high prevalence in ABPA suggests that they may be a risk factor in the development of the disease (Table 4).

### 6.6. IL-10 Polymorphisms

Brouard and coworkers [84] recently reported another genetic risk, the association of the *-1082GG* genotype of the IL-10 promoter with colonization with *A. fumigatus* and the development of ABPA in CF (Table 4). The *-1082GG* polymorphism has been associated with increased IL-10 synthesis; whereas the *-1082A* allele has lower IL-10 synthesis. Thus, dendritic cells expressing HLA-DR2/DR5, increased IL-10 synthesis, and increased sensitivity to IL-4 stimulation due to *IL-4RA* polymorphisms, may be responsible for skewing *Aspergillus-*specific Th2 responses in ABPA.

### 6.7. Surfactant Protein A2 (SP-A2) Polymorphisms

Recently, Saxena et al. [85] reported that ABPA patients with polymorphisms (ala91pro, arg94arg) in the collagen region of pulmonary surfactant protein A2 (*SP-A2*) had more elevated total IgE levels and higher percentages of eosinophilia than observed in those patients who lacked the SNPs (Table 4). They also found that 80% of patients carrying both alleles had ABPA (*P* = .0079, OR = 10.4), while only 50% and 60% of patients carrying each allele, individually, were ABPA subjects, suggesting an additive effect. How these SNPs affect SP-A has not yet been elucidated, but the collagen region spanning both SNPs has been shown to associate with receptors of alveolar macrophages [86], which are important in protecting against *Aspergillus* colonization [32]. It is theorized that changes in conformation or affinity of SP-A2 may decrease these interactions and compromise host defense.

### 6.8. Cystic Fibrosis Transmembrane Conductance Regulator (*CFTR*) Gene Mutations

Because ABPA is found in highest incidence among atopic patients with CF, Miller et al. [87] examined mutations in the cystic fibrosis transmembrane conductance regulator gene (*CFTR*) in subjects without CF (Table 4). Their group reported that mutations were present at a higher frequency in asthmatic patients who developed ABPA, 6 of 21 (28.5%), versus control asthmatics, 2 of 43 (4.6%). These ABPA patients were heterozygous for the mutations (1 patient was compound heterozygote and reclassified as atypical CF), did not have a clinical diagnosis of CF, and had sweat chlorides <60 mEq/L. Although the abnormal airway mucus in CF is thought to be a susceptibility factor for ABPA due to enhanced trapping of *Aspergillus* spores, it is unclear what effect heterozygous *CFTR* mutations may have on mucus quality in asthmatic airways.

### 6.9. Toll-Like Receptor (TLR) Polymorphisms

Wang et al. [88] examined Toll-like receptor (*TLR*) polymorphisms of *TLR2*, *TLR4*, and *TLR9* in cavitary pulmonary aspergillosis (CCPA), and severe asthma associated with fungal sensitization (SAFS). TLR-4 is among the major receptors for *Aspergillus* hyphae and plays an important part in innate host defense as TLR-4 deficient mice have increased susceptibility to invasive aspergillosis [88]. In CCPA patients, there was significantly increased frequency of the G allele of *TLR4* on asp299gly. ABPA patients had increased frequency of allele C for the *TLR9 *T-1237C polymorphism compared to control patients. However, in SAFS patients who are predominantly *Aspergillus* sensitive, there was no association of polymorphisms of *TLR2*, *TLR4*, or *TLR9*. TLR-9 is a receptor that recognizes CpG motifs prevalent in bacterial and viral DNA. *Aspergillus* hyphae and conidia do signal through TLR-9 on murine neutrophils [89]. TLR-9 deficient mice demonstrate greater conidial and hyphal damage. In addition, Lazarus et al. [90] reported that TLR9 polymorphisms have been associated with increased risk of asthma. Novak et al. [91] reported that the *TLR9* C allele of T-1237C decreases expression. Thus, decreased TLR-9 protective function may be an underlying susceptibility in the development of ABPA.

There are additional genetic risk factors in the development of allergy to fungi that include *integrin β3* (*ITGB3)* polymorphisms and chitinase polymorphisms. However, they have not been examined in ABPA.

### 6.10. Integrin *β*3 (ITGB3)

*ITGB3* encodes a *β*-integrin that comprises part of the platelet and monocyte specific heterodimeric receptor for fibrinogen and the receptor for vitronectin. Gerold et al. [92] reported that vitronectin specifically bound triacylated lipopeptides. Vitronectin facilitates the delivery of lipopeptides to the vitronectin receptor which is part of the TLR-2 activation complex. Vitronectin binds to 6 different integrin receptors by an arg-gly-asp (RGD) motif; the main receptor, though, is integrin *α*v*β*3 (integrin *β*3). Recently, Weiss et al. [93] reported single nucleotide polymorphisms (SNP) of *ITGB3* associated with asthma and sensitization to mold allergens. Bromley and Donaldson [94] demonstrated that the RGD peptide sequence is the ligand for integrin *β*3. Whether this polymorphism is a risk factor in the development of ABPA needs to be examined.

### 6.11. Chitinases

Chitin is a major structural component of the outer coatings of many organisms such as fungi, parasitic nematodes, and arthropods [95, 96]. Chitin has been shown to skew the immune system towards Th1 response by suppressing Th2-mediated IgE production and lung eosinophilia in allergic mice. In humans, acidic mammalian chitinase degrade these chitins, thereby shifting the response toward a Th2 inflammatory response. Elevated chitinase has been associated with asthma and elevated IgE levels perhaps through an IL-13 pathway [97, 98]. Furthermore, polymorphisms in the promoter of acidic mammalian chitinase have been associated with atopic asthma and elevated IgE levels. Their role in ABPA is yet to be determined.

### 6.12. Adhesion Molecules

Mutation of a number of adhesion molecules has been associated with asthma and decline of pulmonary function. However, they have not been studied for association with sensitivity to *A. fumigatus*, colonization with *A. fumigatus*, or development of ABPA. These include A disintegrin and metalloprotease 33 gene (*ADAM33*), filaggrin (*FLG*), protocadherin 1 (*PCDH1*), plasma urokinase plasminogen activator (*PLAUR*), and E-cadherin genes. These molecules play an important role in maintaining the integrity of the airway epithelium. Kauffman's group reported that *A. fumigatus* and other fungal proteases disrupt bronchial epithelial integrity [30, 31]. Furthermore, Fairs et al. [17] identified that IgE sensitization to *A*. *fumigatus* and colonization with *A. fumigatus* in asthmatics was associated with a more rapid decline of pulmonary function. Further studies are needed to examine whether *A. fumigatus* proteases are more damaging to bronchial epithelial integrity when mutations of these adhesions molecules are present.

### 6.13. A Disintegrin and Metalloprotease 33 Gene (ADAM33)

ADAM33 is a member of the ADAM family, which is involved in cell adhesion, cell fusion, cell signaling, and proteolysis. ADAM33 is expressed on airway smooth muscle cells and fibroblasts [99, 100]. It has been proposed to contribute to airway remodeling in asthma. SNPs of ADAM33 have been associated with reduced lung function and rapid decline of FEV-1 in asthma.

### 6.14. Filaggrin (FLG)

Filaggrin contributes to the skin barrier and mutations of FLG have been associated with eczema. In addition, FLG SNPs have been associated with the development of asthma [101].

### 6.15. Protocadherin 1 (PCDH1)

Protocadherins play an important role in cell adhesion and organ development. Protocadherins 1 is expressed in the apical adhesion complex of airway epithelia cells. It is postulated that PCDH1 may be important in epithelial integrity of the airways. Koppelman et al. [102] reported that polymorphisms of *PCDH1* play a role in the development of bronchial hyperresponsiveness in asthma.

### 6.16. Plasma Urokinase Plasminogen Activator (PLAUR)

PLAUR may play a role in the pathogenesis of airway remodeling in asthma. Barton et al. [103] reported that SNPs of PLAUR are associated with development of asthma and lung function decline in asthma.

### 6.17. E-Cadherin

E-cadherin forms cell-cell contacts promoting integrity of the airway epithelium [104]. Reduction of E-cadherin is observed during airway responses and results in increased epithelial permeability and increased airway hyperresponsiveness.

## 7. Treatment

Treatment is designed first to control the acute episodes and then to limit the development of chronic lung disease [19–23]. Most cases of ABPA require treatment with systemic corticosteroids, and the treatment of choice is prednisone. Steroid therapy rapidly clears the eosinophilic infiltrates and the associated symptoms, although it is less effective at treating mucus impaction. In asthmatic ABPA patients, the usual starting dose is 0.5 mg/kg/day, taken each morning, and this dose is maintained for 2 to 4 weeks while following the patient clinically and checking the chest radiograph for resolution of the acute process. After this induction treatment, the dose of prednisone should be reduced to 0.5 mg/kg given on alternate days. If mucus impaction persists and is associated with atelectasis, bronchoscopy should be performed to confirm the diagnosis and to attempt to remove the mucus plugs. Following resolution of the acute process, the dose of prednisone should be reduced over 1 to 3 months. Chronic treatment with corticosteroids is controversial, especially in adults, because only minorities of patients with ABPA are at risk of chronic lung disease. The relationship between acute episodes and lung damage is unclear, and the precise dose of prednisone is not certain, since acute exacerbations may continue while the patients are on low doses of steroids. 

Children with ABPA usually have CF and may need treatment with higher corticosteroid dosing and long-term corticosteroids to prevent progressive lung damage. Stevens et al. [23] in the Cystic Fibrosis Foundation Consensus Conference report recommended in ABPA CF patients an initial dose of 2 mg/kg/day of prednisone for 1 week. This is then reduced to 1 mg/kg/day for 1 week that was followed by reducing to alternate day dosing. This prednisone dose is then gradual tapered typically. We usually maintain therapy with a dose of 0.5 mg/kg on alternate days for 3 months and then, after 3 months, the dose of prednisone is tapered over a further 3 months while checking the chest radiograph and the serum IgE level for evidence of relapse. Initially, the serum IgE level should be checked at every visit and, if the level increases by twofold or more, the steroid dose should be increased. We recommend that patients are followed with serum IgE levels and chest radiographs every 6 months for the first 1 to 2 years, and then, if the child remains in remission, it should be possible to reduce the frequency of these studies. Unfortunately, this high and prolonged corticosteroid treatment may induce diabetes in these CF patients. Treatment with itraconazole and omalizumab should be considered as adjunctive treatment of ABPA (see below). Furthermore, their use may be helpful in preventing exacerbation of ABPA in CF patients. Monitoring of ABPA in CF patients is done at multiple levels. These include clinical symptoms of dyspnea, cough and wheezing, pulmonary function studies, serum IgE levels, IgE and IgG specific anti-*A. fumigatus* antibodies, blood eosinophilia, and blood glucose.

The antifungal agent itraconazole has been used to reduce the doses of steroids that are required [105, 106]. Initially, there were only open nonrandomized studies that indicated that itraconazole is a useful adjunct to systemic corticosteroid therapy. Two recent randomized controlled trials have also favored intraconazole use. A double-blind, randomized, placebo-controlled trial of itraconazole 200 mg twice daily dose resulted in decreased IgE levels and an increase in pulmonary function and exercise tolerance. Another randomized, controlled trial showed that treatment of stable ABPA in adults with 400 mg/day itraconazole resulted in a significant reduction in sputum eosinophil count, sputum eosinophilic cationic protein levels, serum IgE concentrations, and *Aspergillus*-specific IgG. There was also a reduction in episodes of exacerbation requiring treatment with systemic steroids. In the treatment of children with ABPA, we have used a dose of 10 mg/kg/day of itraconazole. Omalizumab, anti-IgE monoclonal antibody, has been used in uncontrolled reports. Anecdotally, it has been effective, but a randomized controlled trial is necessary.

There is no place for immunotherapy in children with ABPA, since it is ineffective and potentially dangerous. Inhaled anti-inflammatory agents, such as cromoglycate and corticosteroids are not generally thought to be effective. The role of inhaled spores in the pathogenesis of ABPA is unclear, but there is a seasonal incidence of ABPA that is probably related to seasonal changes in mold spore counts. Therefore, it is reasonable to advise patients with ABPA to avoid exposure to places with high spore counts, such as damp basements, barns, and compost heaps.

## 8. Prognosis

The prognosis of ABPA is good if the disease is detected early and treatment started promptly. It is important that the diagnosis is made and treatment commenced before there is permanent lung damage from bronchiectasis. In such patients, there should be no progression of the disease, although relapses can occur many years later, and long-term followup is recommended. In children with CF, the relapses seem to be more frequent than they are in patients with asthma, and careful surveillance is necessary to ensure resolution of the disease process. In some CF patients, it is difficult to wean the steroids without an increase in symptoms, such as dyspnea and wheezing; whether this is due to the underlying CF lung disease or due to patients going from stage II to stage III ABPA on withdrawal of steroids is unclear. Symptoms are not a reliable guide to therapy; therefore, it is important to reevaluate the chest radiograph and the serum IgE at regular intervals until a long-term remission is established.

## 9. Conclusions

ABPA occurs with a world-wide distribution in a significant number of patients with CF and less frequently in those with asthma. Early diagnosis and treatment are essential in preventing end-stage progression. The development of ABPA is probably the combination of many genetic susceptibility factors, gene-gene interactions, and environmental exposure which work together. Understanding of the genetic risks and immunopathogenesis of ABPA hopefully will lead to improved early diagnosis and improved treatment of ABPA.

## Figures and Tables

**Table 1:** Criteria for diagnosis of allergic bronchopulmonary aspergillosis in asthma.

(1) Asthma	
(2) Chest radiographic infiltrate(s)	
(3) Allergy prick skin reactivity to *A. fumigatus *	
(4) Elevated total serum IgE level ≥1000 IU/mL. Some groups recommend IgE ≥1000 ng/mL (416 IU/mL)	
(5) Precipitating IgG antibodies to *A. fumigatu*s	
(6) Peripheral blood eosinophilia	
(7) Elevated serum specific IgE anti-*A. fumiatus* antibodies greater than twice non-ABPA IgE *A. fumigatus*-positive asthmatic serum pool	
(8) Elevated serum specific IgG anti-*A. fumiatus* antibodies	
(9) Central bronchiectasis	

(i) Criteria 1–9, ABPA-central bronchiectasis, ABPA-CB.

(ii) Criteria 1–8, ABPA-seropositive, ABPA-S.

**Table 2:** Criteria for diagnosis of allergic bronchopulmonary aspergillosis in cystic fibrosis.

*Classic Diagnostic Criteria*	
(i) Acute or subacute clinical deterioration not attributable to another etiology	
(ii) Total serum IgE concentration greater than 1000 IU/mL unless patient is receiving corticosteroid therapy	
(iii) Immediate cutaneous reactivity to *Aspergillus fumigatus* while the patient is not being treated with antihistamines or *in vitro* presence of serum IgE antibody to *A. fumigatus *	
(iv) Precipitating antibodies or serum IgG antibody to *A. fumigatus *	
(v) New or recent abnormalities on chest radiography or chest CT that have not cleared with antibiotics and standard physiotherapy	

*Minimal Diagnostic Criteria*	
(i) Acute or subacute clinical deterioration not attributable to another etiology	
(ii) Total serum IgE concentration greater than 500 IU/mL unless patient is receiving corticosteroid therapy. If ABPA is suspected and the total level of 200 to 500 IU/mL, repeat testing in 1 to 3 months is recommended. If patient is taking steroids, repeat when steroid treatment is discontinued.	
(iii) Immediate cutaneous reactivity to *Aspergillus fumigatus* while the patient is not being treated with antihistamines or in vitro presence of serum IgE antibody to *A. fumigatus *	
(iv) One of the following: (a) precipitins to *A. fumigatus* or in vitro documentation of IgG antibody to *A. fumigates,* or (b) new or recent abnormalities on chest radiography or chest CT that have not cleared with antibiotics and standard physiotherapy	

**Table 3:** Staging of allergic bronchopulmonary aspergillosis in asthmatics.

Stage	IgE level	Precipitins	Eosinophilia	IgE-Af	IgG-Af	Pulmonary infiltrates
(I) Acute	+++	+	+	+	+	+
(II) Remission	+	±	−	±	±	−
(III) Exacerbation	+++	+	+	+	+	+
(IV) Corticosteroid dependent	++	±	±	±	±	−
(V) Fibrotic	+	±	−	±	±	−

**Table 4:** Genetic risk factors in the development of allergic bronchopulmonary aspergillosis.

(i) HLA-DR restriction and HLA-DQ protection	
(1) HLA-DR2	
(a) HLA-DRB1*1501 and *HLA-DRB1*1503	
(2) HLA-DR5	
(3) HLA-DQ2 protective, decreased in ABPA	
(a) DQB1*0201	
(ii)* IL-4RA* polymorphisms	
(1)* IL-4RA* ile75val	
(iii) IL-10 polymorphisms	
(1) Promoter -1082 GG genotype	
(iv) Surfactant protein A2 (*SP-A2*) polymorphisms	
(1)* SP-A2* ala91pro	
(v) Cystic fibrosis transmembrane conductance regulator (*CFTR*) mutations	
(1) Heterozygous *CFTR* mutations in asthmatic patients with ABPA	
(vi) Toll-like receptor (*TLR*) polymorphisms	
(1)* TLR9 *T-1237C polymorphism	

## Abbreviations

ABPA: Allergic bronchopulmonary aspergillosis
Af: *Aspergillus fumigatus*
*CFTR*: Cystic fibrosis transmembrane conductance regulator
*IL-4RA*: IL-4 receptor alpha chain
TARC: Thymus and activation-regulated chemokine
*SP-A2*: Surfactant protein A2 polymorphisms.
